# Electrophysiological evidence of Hebbian plasticity in awake adult rats

**DOI:** 10.1152/jn.00129.2025

**Published:** 2025-09-15

**Authors:** Siddharth S. Gaikwad, Yi Chen, Bing Chen, Wil H. D. Bogue, Giuseppe Scesa, Matthew S. Neehouse, Theresa M. Vaughan, Jonathan S. Carp, Martin Oudega, Jonathan R. Wolpaw, Monica A. Perez

**Affiliations:** 1 Shirley Ryan AbilityLab, Chicago, Illinois, United States; 2 National Center for Adaptive Neurotechnologies, Albany Stratton VA Medical Center, Albany, New York, United States; 3 Department of Physical Medicine and Rehabilitation, Northwestern University, Chicago, Illinois, United States; 4 Department of Biomedical Sciences, State University of New York, Albany, New York, United States; 5 Department of Physical Therapy and Human Movement Sciences, Northwestern University, Chicago, Illinois, United States; 6 Department of Neuroscience, Northwestern University, Chicago, Illinois, United States; 7 Edward Hines Jr. VA Hospital, Hines, Illinois, United States

**Keywords:** H-reflex, motor-evoked potentials, neural plasticity, neuromodulation, STDP

## Abstract

Hebbian stimulation, based on principles of spike timing-dependent plasticity (STDP), has been successfully used to enhance functional recovery in individuals with spinal cord injury (SCI). To advance therapies using Hebbian stimulation, this study aimed to establish STDP-based protocols targeting spinal motoneuron synapses in awake rats. Adult male and female Sprague–Dawley rats were implanted with stainless steel screws through the skull over the hindlimb area of the left motor cortex to enable epidural cortical stimulation. A custom-made cuff with embedded fine-wire electrodes was placed around the right posterior tibial nerve for peripheral stimulation. Fine-wire electrodes were inserted in the soleus muscle to record motor-evoked potentials (MEPs), H-reflexes, and the maximal motor response. During Hebbian stimulation, descending volleys evoked by cortical stimulation were timed to reach spinal motoneurons either 2.5 ms before (Hebbian+) or 15 ms after (Hebbian−) the arrival of antidromic potentials evoked by tibial nerve stimulation on different days. Rats received 180 paired pulses over 30 min, with measurements taken at baseline and every 10 min up to 40-min poststimulation. We found that MEP size increased by an average of 30% over baseline during the 40-min poststimulation period with Hebbian+ stimulation and decreased by an average of 27% with Hebbian− stimulation. These findings provide the first evidence that paired stimulation based on Hebbian STDP principles can bidirectionally modulate MEPs in awake rats. Our rat model of Hebbian stimulation paves the way for exploring experimental combination therapies to enhance motor recovery following SCI and other neurological disorders.

## NEW & NOTEWORTHY

Hebbian stimulation, based on spike timing-dependent plasticity (STDP), has been used to enhance functional recovery in spinal cord injury (SCI). Here, we show that paired stimulation following STDP principles can bidirectionally modulate motor-evoked potential size in the soleus muscle of awake rats. This rat model provides a valuable platform for exploring combination therapies to improve motor recovery in SCI and other neurological disorders.

## INTRODUCTION

Over the past few decades, neurostimulation has emerged as a promising strategy to enhance sensorimotor function and improve the quality of life for individuals with spinal cord injury (SCI) (1–3). One such approach, Hebbian stimulation, is based on the principles of spike-timing-dependent plasticity (STDP), which relies on the precise timing and sequence of action potential arrival between presynaptic and postsynaptic neurons (4, 5). In humans, Hebbian stimulation has been applied to corticospinal-motoneuronal synapses that contribute to the control of both single and multiple limb muscles (6–8). Placebo-controlled clinical trials have shown that this approach can lead to significant functional recovery in individuals with SCI (8, 9), with even greater improvements observed when Hebbian stimulation is combined with other therapies that further enhance plasticity (10). Extending Hebbian stimulation to awake, behaving rat models could provide valuable insights into its underlying mechanisms and offer opportunities to test therapies that may further amplify its functional benefits following SCI.

Several studies have investigated the effects of paired stimulation or paired associative stimulation−an experimental approach that leverages Hebbian timing rules to induce neuroplasticity−in the spinal cord of rats. However, interpreting the underlying mechanisms remains challenging (11–17). A key limitation is that estimates of action potential arrival times from both central and peripheral origins at the spinal cord level are often random, lacking precise calculations of central (CCT) and peripheral (PCT) conduction times (11–17). Hebbian plasticity depends on the ability to induce either facilitatory or inhibitory effects, determined by the timing of action potential arrival at the targeted synapse (8, 9). Several of these studies have applied trains of pulses to various stimulation sites to evoke facilitatory effects (11, 13–17), making it difficult to elucidate the underlying mechanisms involved. These studies likely rely on a convergent model of plasticity, where synaptic changes result from the summation of temporally paired inputs originating from multiple presynaptic neurons (18), rather than from the precise pairing of stimulation between a presynaptic and postsynaptic neuron. In addition, many of these studies were conducted under anesthesia (11, 14, 16). Ketamine, a commonly used anesthetic, is known to modulate the amplitude and latency of motor-evoked potentials (MEPs), with effects varying based on anesthetic depth (19). Anesthetics can alter physiological parameters and neuronal plasticity (20–22), potentially influencing study outcomes. Previous animal studies employing paired stimulation protocols—whether adhering to Hebbian timing rules or not—have yielded mixed results. Some studies have found that repeated paired pulses increased MEP size or lowered excitation thresholds (11, 13–17), whereas others found no change (12). Notably, none of these studies have demonstrated a reversible facilitatory and inhibitory effect on MEP size as suggested by Hebbian timing rules and STDP principles (11–17). Here, we hypothesized that applying precisely timed Hebbian stimulation in awake adult rats could either enhance (Hebbian+) or suppress (Hebbian−) MEP size in the soleus muscle.

To test this hypothesis, we examined soleus MEPs elicited by electrical stimulation over the hindlimb representation of the motor cortex in awake, unrestrained adult Sprague- Dawley rats. MEPs were recorded before and after the application of a Hebbian stimulation protocol, in which descending volleys evoked by cortical stimulation were precisely timed to reach the soleus spinal motoneurons either before (Hebbian+) or after (Hebbian−) the arrival of antidromic volleys evoked by posterior tibial nerve stimulation.

## MATERIALS AND METHODS

### Animals

Twelve adult rats were chronically implanted with cortical and peripheral nerve electrodes to receive Hebbian stimulation (Table 1). The implantation methods and the basic recording and stimulation methods have been described in previous studies (23–25) and are summarized here. All procedures were performed in accordance with the Guide for the Care and Use of Laboratory Animals (National Academies Press; Washington, DC), and approved by the Institutional Animal Care and Use Committees at Northwestern University, Chicago, IL and at the Stratton VA Medical Center at Albany, NY. The procedures were performed in Sprague–Dawley rats by surgeons at Northwestern University (*n* = 2, males, 319–359 g; Taconic Biosciences, Germantown, *n* = 5, females, 255–296 g; Strain Code, 001, Charles River Laboratories, Wilmington; referred to as “NU” rats; Table 1) and at the Stratton VA Medical Center (*n* = 5, males, 335–430 g; Taconic Biosciences, Germantown; referred to as “Stratton” rats; Table 1). Implantation surgeries were consistent across the two study sites, with a few differences in the pre- and postsurgery procedures, as described later. Following electrode implantation, all rats were allowed a 3-wk recovery period to stabilize the wound sites before electrophysiological measurements and Hebbian stimulation were initiated (details provided later). MEPs were recorded between 3 and 10 wk (6.4 ± 2.8 wk) after electrode implantation, regardless of weight or gender to measure the effect of Hebbian+ and Hebbian− stimulation (Table 1). For each rat, the Hebbian+ and Hebbian− protocols were applied on separate days in a randomized order. All rats received the Hebbian− protocol, but only 11 received the Hebbian+ protocol, as one animal died during the washout period between protocols. In a few animals, MEPs were recorded between 10 and 22 wk (16 ± 4.4 wk) after electrode implantation as an indicator of electrode stability over time (Table 1).

### Cortical and Peripheral Electrodes

**Figure 1.**
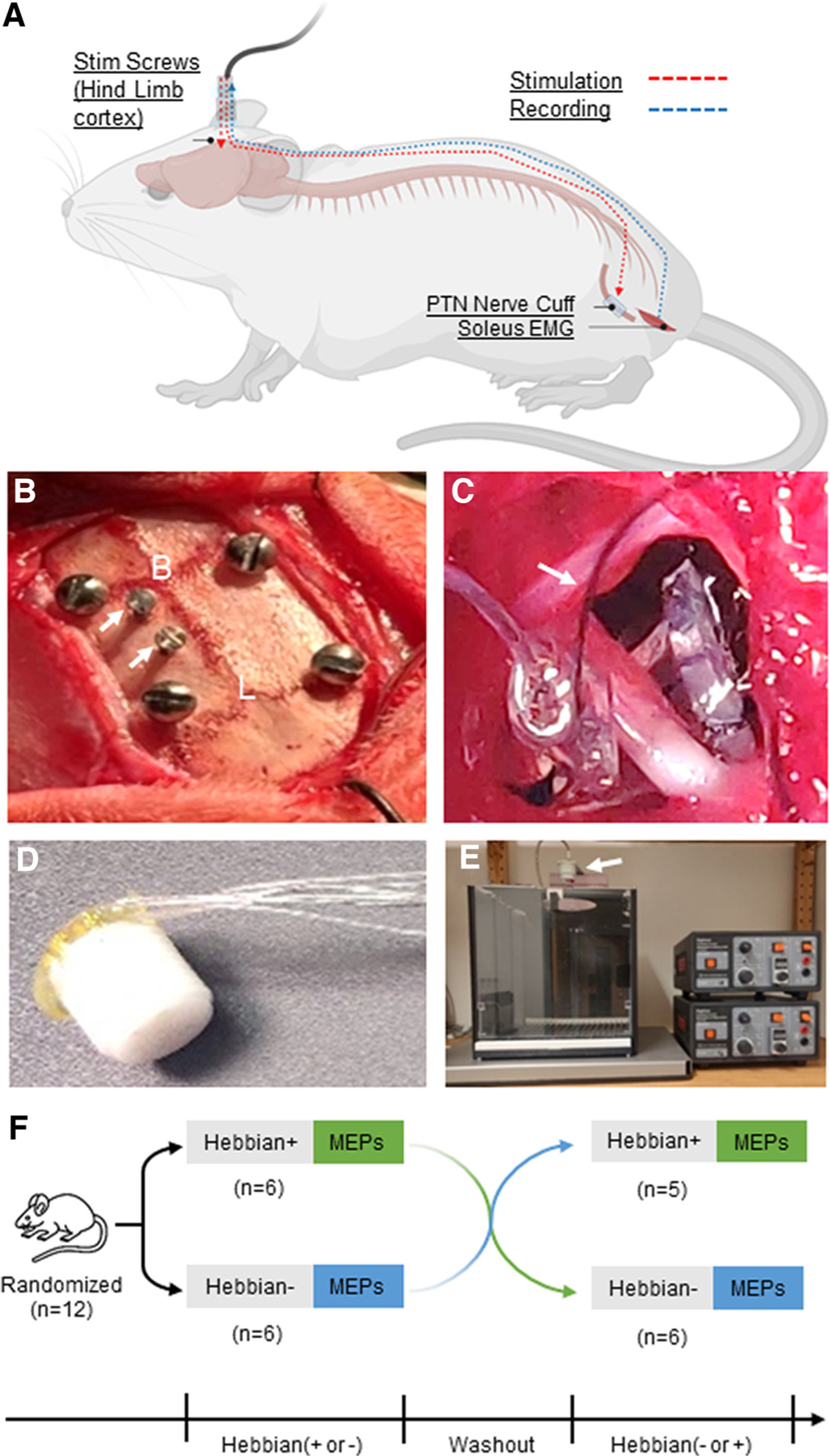
Experimental setup. Surgical implantation of cortical and peripheral nerve electrodes for Hebbian stimulation in adult awake rat. *A*: schematic drawing of adult rat implanted with the cortical and peripheral nerve wire electrodes and head pedestal that can be connected by a cable to the stimulators. With our setup, we can stimulate the hindlimb motor cortex using screw electrodes and the posterior tibial nerve (PTN) using a nerve cuff (red interrupted lines) and record electromyography (EMG) from the soleus muscle (blue interrupted line). *B*: location of four larger corner screws and two smaller electrode screws (white arrows) in the rat’s skull. The larger corner screws serve to anchor the pedestal to the skull using acrylic resin. The smaller electrode screws (arrows) are placed over the hindlimb area of the motor cortex to stimulate the cortical neurons that connect with the spinal cord motoneurons that project their axon through the posterior tibial nerve to the soleus muscle. B, Bregma; L, Lambda. *C*: exposed right posterior tibial nerve just prior to closure of the custom-made silicon cuff. Arrow points to one of the sutures to close the cuff without restricting the nerve. *D*: the head pedestal with inserted and angled wire electrodes. The pedestal is attached to the skull and is connected via a commutator to the stimulation and recording devices. Note that all wires are subcutaneously connecting with the cortical electrodes, the nerve cuff, and the soleus muscle. *E*: stimulation setup with custom-made rat cage with on top the commutator (arrow), and two constant current stimulators (Model DS7AH; Digitimer Ltd., Hertfordshire, UK). *F*: diagram showing the crossover design of the protocols used during Hebbian stimulation. Animals were randomly assigned to 30-min Hebbian+ or Hebbian− protocols and crossover after the washout period. Note that one animal died in the washout period after finishing Hebbian−.

We implanted screw electrodes over the hindlimb representation of the motor cortex and a cuff electrode around the hindlimb posterior tibial nerve to enable stimulation protocols. In addition, a fine-wire electrode pair was inserted into the soleus muscle for recording electrophysiological parameters (Fig. 1A). A custom-made cortical head stage (Fig. 1D) was used, consisting of six 20-cm-long fine stainless steel multi-stranded wires (AS631, Cooner Wire; Chatsworth, CA). The wires were stripped of 4 mm of their Teflon insulation on one end, which was then inserted into a connector pin (E363/0, Plastics One; Roanoke, VA) and soldered. The 20-cm wire length allowed animal movements and growth while providing flexibility for adjustments during the fabrication of the nerve cuff and soleus electromyographic (EMG) electrodes. All six pins were inserted into a small plastic pedestal (MS363, Plastics One; Roanoke, VA), and the wires were gently bent 90° just before entering the pins (Fig. 1D). The exposed pins, solder joints, and the first few millimeters of wire were encased in epoxy. Of the six wires leaving the pedestal, two were stripped of insulation at the ends and fitted 2 mm apart in the custom-made silicone nerve cuff (length: 4 mm; lumen: 2 mm) for posterior tibial nerve stimulation. Two wires were cut to 10 cm, stripped of insulation for the final several centimeters, and connected to the epidural screws used for cortical stimulation. The remaining two wires were stripped of insulation for the final centimeters and inserted into the soleus muscle to record EMG activity. The nerve cuff was secured with 6-0 nylon sutures (Ethicon, NJ, USA) to gently close around the tibial nerve without causing compression. Before implantation, the integrity and connectivity of the assembled implants were verified using a Fluke 77-IV digital multimeter (Everett, WA).

### Preparation for Implantation Surgery

Rats used at the Stratton VA were anesthetized with ketamine (80 mg/kg ip; Mylan Pharmaceuticals, Canonsburg, PA) and xylazine (10 mg/kg ip; Akorn Inc., Lake Forest, IL), which was supplemented as needed (23–25). After deep surgical anesthesia was induced, each rat was given preoperative analgesia of buprenorphine (0.03 mg/kg sc; McKesson, Irving, TX) and carprofen (5 mg/kg sc; Ceva Animal Health, LLC, Lenexa, KS). Head and right hindlimb were shaved and cleaned with Nolvasan skin (Zoetis, Parsippany, NJ), and eyes were treated with Refresh Lacri-Lube ointment (Allergan, Madison, NJ) to prevent drying (23–25). The anesthetized rat was fixed in a rat stereotaxic apparatus with the head leveled and secured with standard ear bars and a tooth holder, and the right hind limb was fixed with waterproof adhesive tape to custom-made ridges on the stereotaxic apparatus. The surgical site was kept accessible using metal hooks and rubber bands attached to custom-made ridges on the stereotaxic apparatus (23–25). Rats at Northwestern University underwent the same surgical preparation as the rats at the Stratton VA, with the only difference that they were anesthetized with ketamine (60 mg/kg ip; Butlerschein, Dublin, OH) and dexmedetomidine (0.5 mg/kg ip; Pfizer, New York, NY) (26).

### Electrode Implantation Surgery

There were no differences in the electrode implantation surgery between the two study sites. A 2-cm-long incision was made along the rostral-caudal midline of the skull, and a 4-cm-long incision was made from the posterior mid-thigh to the distal end of the knee on the right hind limb. The skin edges were gently retracted using surgical hooks to expose the underlying skull and leg muscles, respectively (Fig. 1, B and C). Next, a hemostat was inserted through the leg skin incision and gently maneuvered subcutaneously, exiting through the skull skin incision. The free ends of two electrode wires and the nerve cuff with its two wires were carefully grasped with the hemostat, which was then slowly withdrawn, allowing the four wires and the nerve cuff to emerge through the leg skin incision. The remaining two electrode wires and the pedestal stayed at the skull skin incision.

To implant the cortical stimulation electrodes, four burr holes were drilled at the corners of a 1 × 2-cm rectangle on top of the skull. Stainless steel screws (0–80 × 3/32 in., 2.4-mm long) were placed in the four holes with their heads ~1 mm above the surface of the skull (Fig. 1D). Next, two small burr holes were drilled into the left skull 2.8-mm lateral to the mid- line and 1 mm and 3 mm caudal to Bregma, which is above the right hindlimb cortical representation. Two 0.5-mm diameter stainless steel screws (J.I. Morris Miniature Fasteners, Oxford, MA) were gently secured in these holes with their heads ~0.5 mm above the skull (Fig. 1B). The two cortical stimulation wires were cut to 5–10 cm length; their ends were stripped of insulation and wrapped around the two 0.5-mm diameter screws. The screws were then further tightened gently to secure the cortical stimulation wires around the screws and on top of the skull.

To implant the peripheral nerve cuff, the posterior tibial nerve was exposed by blunt dissection (Fig. 1C). The nerve cuff was opened along a longitudinal slit to allow it to be placed gently around the nerve without disturbing the nerve anatomy or location (Fig. 1C). The nerve cuff was closed around the nerve using a pair of 6–0 nylon sutures. To record soleus EMG activity, the two EMG electrode wires were fed into the point of a 21-G needle (width: 0.8 mm; length: 25 mm), which was then inserted just above the ankle into the caudal portion of the soleus muscle and advanced to its rostral end. The two uninsulated wire ends were offset from each other so that they would not short out. The needle was then gently withdrawn while turning counterclockwise, thereby ensuring that the uninsulated ends of the two electrodes remained within the muscle. The surgical hooks were removed and the muscles at the leg incision site were gently maneuvered back into their natural position and closed with 5-0 absorbable sutures. The wound site was cleaned, injected with 0.5% bupivacaine hydrochloride (Hospira, Inc.), and closed with 9-mm EZ Clips wound clips (Stoelting).

After all electrodes were implanted, the electrode pedestal was anchored to the four larger skull screws with acrylic resin (Ortho-Jet BCA package, Lang dental, Wheeling, IL). The surgical hooks were removed, and the skin was repositioned over the skull and around the acrylic resin. Then, the wound site was cleaned as described earlier and closed with 9-mm EZ Clips wound clips (Stoelting), thereby ensuring a snug fit of the skin around the pedestal.

### Postsurgery Procedures

All rats survived the surgery and were closely monitored until fully awake. Rats at Stratton VA received a topical application of antimicrobial Nitrofurazone ointment (FURA-zone) at the surgical sites and were provided with dietary supplements (e.g., Nutri-Cal, apple) until they regained their presurgery body weights. All rats were housed individually under a 12-h light/dark cycle with ad libitum access to food and water, and they were monitored daily (23–25). The postsurgery care at Northwestern University was similar, with the key difference being that rats received Antisedan (1 mg/kg sc; Pfizer) to reverse anesthesia, and no dietary supplements were provided (26).

### Stimulation and Recording Procedures

The electrodes were connected to external devices through a six-wire flexible cable (Plastics One) attached to the skull-mounted pedestal and to a commutator above the cage (Fig. 1E). For stimulation of the cortex and the posterior tibial nerve, the wires from the screws implanted in the skull over the right hindlimb motor area and from the nerve-cuff electrodes, respectively, were connected to constant current stimulators (Model DS7AH, Digitimer Ltd., Hertfordshire, UK) (Fig. 1E). This setup allowed implementing Hebbian stimulation protocols (Fig. 1F). For recording soleus EMG, the intra-muscular electrodes were connected to a pre-amplifier (NL844 AC Pre-amplifier, Digitimer Ltd., Hertfordshire, UK). The signals were amplified, filtered (20–1,000 Hz), and sampled at 4 kHz for offline analysis (CED 1401 with Signal V 7.02 software, Cambridge Electronic Design, Cambridge, UK).

### Hebbian Stimulation

Hebbian stimulation protocols were applied ~16 wk after electrode implantation. During paired stimulation, one pair of stimuli was given every 10 s for 30 min (0.1 Hz) for a total of 180 paired stimuli using Signal V 7.02 software (Cambridge Electronic Design, Cambridge, UK). Based on the interstimulus interval (ISI) between the cortical and peripheral stimuli used in humans (6, 7, 27), we ensured that cortical descending volleys arrived at the presynaptic terminals either 2.5 ms before (Hebbian+) or 15 ms after (Hebbian−) the peripheral antidromic volleys reached the spinal motoneurons.

**Figure 2.**
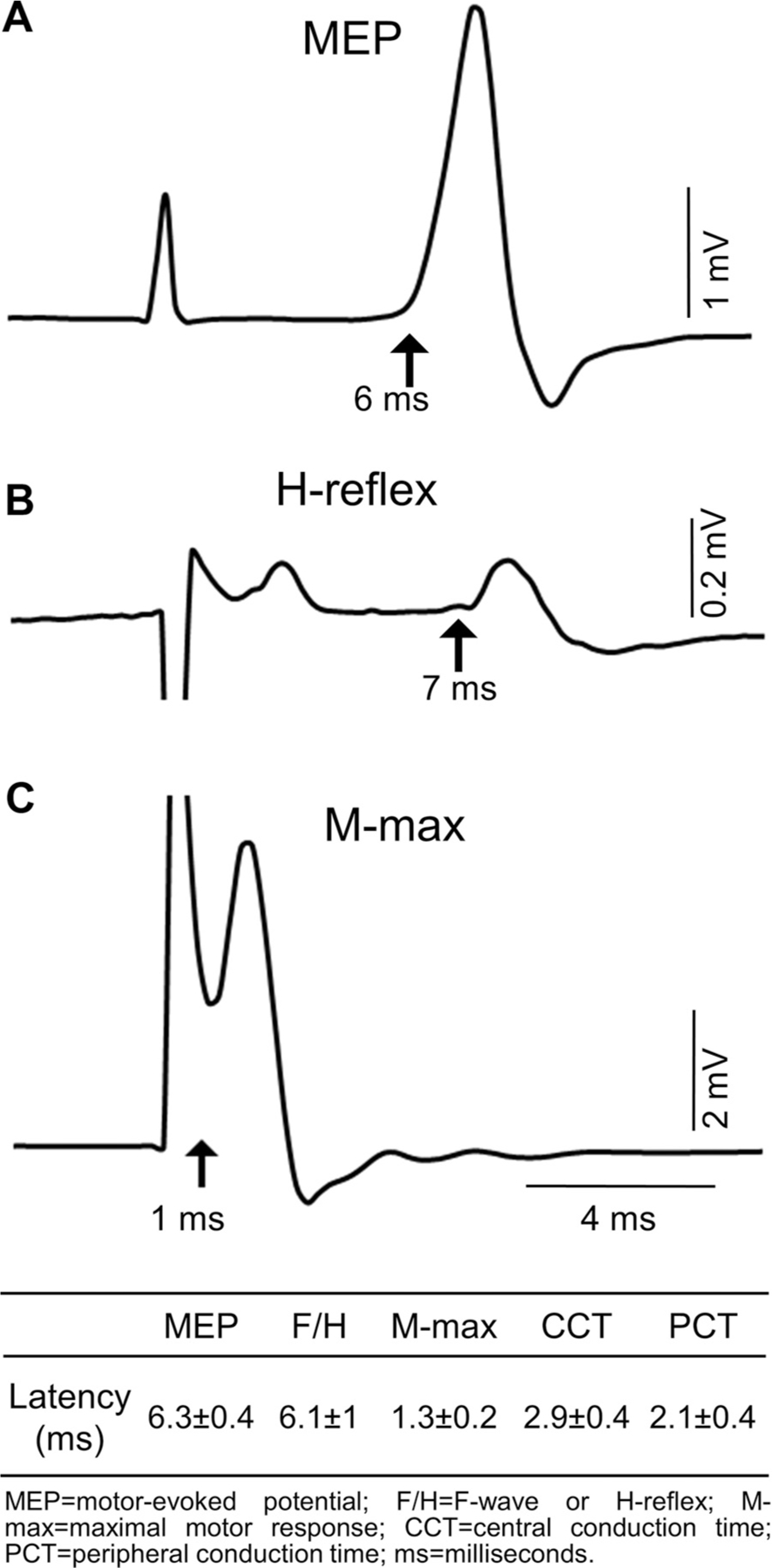
Central (CCT) and peripheral (PCT) conduction time. Representative raw traces showing a motor-evoked potential (MEP) (*A*), and H-reflex (*B*), and a maximal motor response (M-max) (*C*) in an awake adult rat with the implanted cortical and peripheral electrodes. The waveforms represent an average of 20 responses. The black arrow indicates the latency of each response. The latency of MEPs, H-reflexes, F-waves, and M-max were used to determine CCT and PCT, which are needed to define Hebbian protocols. ms, milliseconds; mV, millivolts.

The methods for determining the ISI to properly time the arrival of the central and peripheral volleys at the spinal cord motoneuron synapses were described previously (6, 7, 27). The ISI used during Hebbian+ and Hebbian− protocols was tailored to individual rats based on conduction times calculated from latencies of MEP (Fig. 2A), H-reflex (Fig. 2B), F-wave, and the maximal motor response (M-max; Fig. 2C). The onset latency for each response was defined as the time in the average waveform when the response exceeded 2 standard deviations (SD) of the mean rectified activity for 100 ms prestimulation. The PCT was calculated using the equation, PCT = (H-reflex latency – M-max latency-1) × 0.5. The CCT was calculated using the equation, CCT = MEP latency – (PCT + M-max latency). To ensure optimal stimulation parameters during Hebbian stimulation protocol implementation, the minimum intensities capable of generating maximal MEPs and M-waves were identified for each rat (Fig. 2) and used in the study. This individualized approach allowed precise cortical and peripheral stimulation during the intervention; it was tailored to each rat’s specific neurophysiological profile.

### MEPs

The maximal MEP for the right soleus muscle was deter- mined in each of the rats (Fig. 2A). The maximal MEP was defined at rest by increasing stimulus intensity until the MEP amplitude did not further increase. During measurements before and after the different Hebbian stimulation protocols, the intensity was set at the minimum intensity sufficient to evoke maximum MEP. Pulses were delivered at 4-s intervals (0.25 Hz). In total, 20–30 MEPs were elicited, and their peak-to-peak MEP amplitudes were averaged. To ensure that the MEPs assessed were elicited in the presence of comparable soleus background EMG activity for the 100 ms preceding stimulation, trials in which background EMG activity was higher than 2 SD of mean resting background EMG activity were excluded from analysis [3.9 ± 2.9% in Hebbian+, and 3.1 ± 2.5% in Hebbian− (7, 27)]. We followed up the effects of Hebbian+ and Hebbian− stimulation in a subset of animals (*n* = 3) and found that MEP size returned to baseline ~60 to 120 min poststimulation. This finding is consistent with previous reports on rats (11, 13) and humans (7, 27, 28).

### Data Analysis

Distribution normality was tested using the Shapiro–Wilk’s test and homogeneity of variances by the Levene’s test. When normality was not met, the natural log transformation was used. Sphericity was tested using Mauchly’s test. When sphericity was not met, the Greenhouse–Geisser correction was used. Repeated-measures ANOVA was used for power and sample size calculation using the dependent variable of MEPs (mean diff = 51.4%, SD = 11.8%). With a partial $\eta^{2}\left(\eta_{\text{p}}^{2}\right)$ of 0.378, we tested our hypothesis at α 0.05 and with power of 95% (0.95). A total of 12 rats were needed to achieve a significant difference in our primary outcome measure. A repeated-measures ANOVA with the within-subjects factor TIME (baseline, 10, 20, 30, and 40 min) was used to assess the effect on background EMG and MEP size for each Hebbian protocol. In addition, repeated-measures ANOVAs including the within-subjects factor TIME and the between-subjects factors SOURCE (Taconic/Stratton, Charles/NU) or SEX (female, male) were used to determine their effects on MEP size for each Hebbian protocol. Bonferroni tests were used post hoc to assess significant comparisons. Correlation on MEP size between Hebbian+ and Hebbian− protocol was done using Pearson’s linear correlation coefficient. All statistical analyses were conducted using SPSS (IBM Corp. Released 2019. IBM SPSS Statistics for Windows, v.26.0. Armonk, NY: IBM Corp) with the significance set at *P* < 0.05. Effect sizes of the significant F test results are presented using $\eta_{\text{p}}^{2}$. Group data are presented as means ± SD in the text.

## RESULTS

### Hebbian+

**Figure 3.**
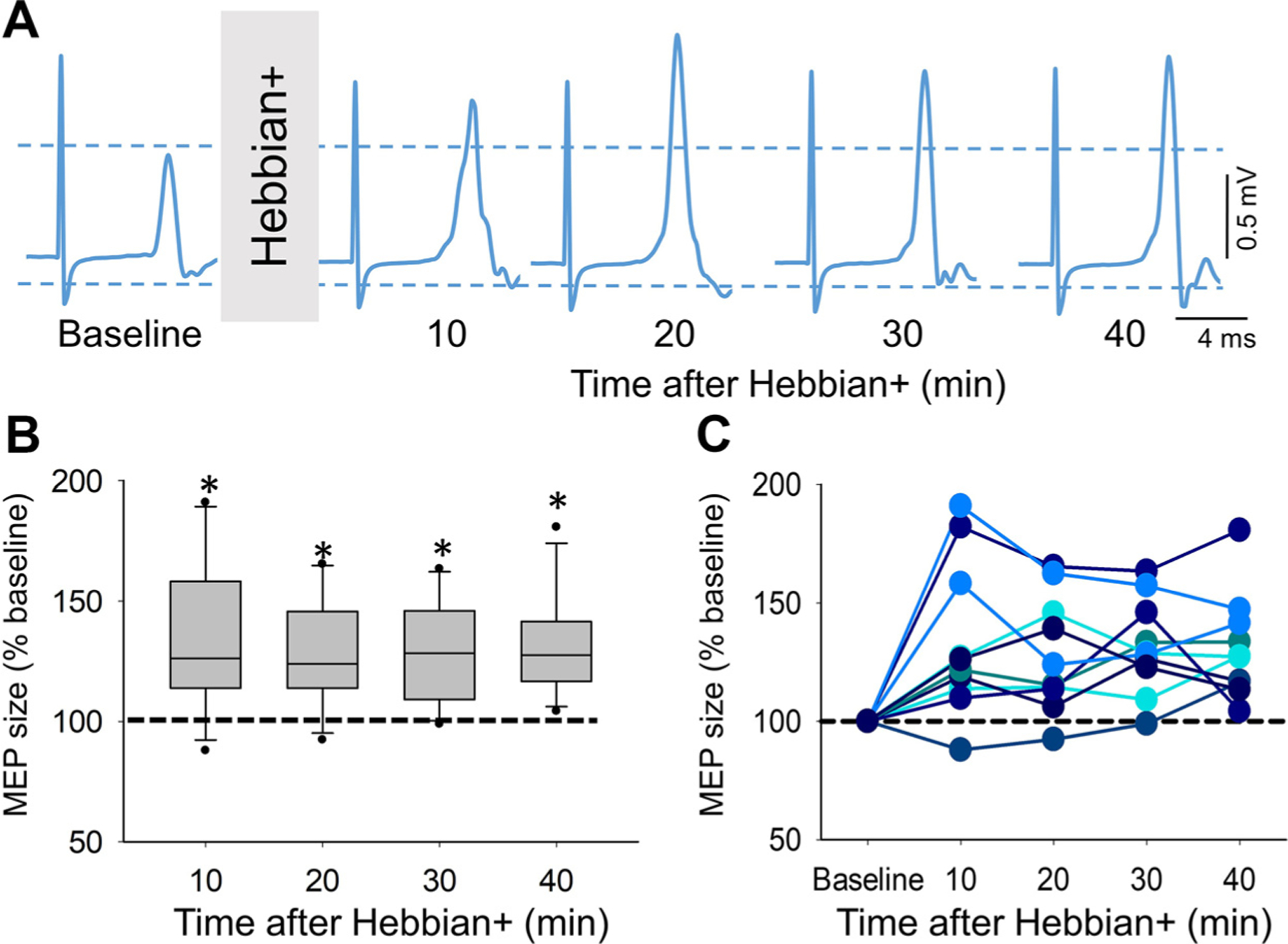
Hebbian+ (*A*). Representative raw motor-evoked potential (MEP) traces from the soleus muscle in an awake adult rat recorded before (baseline) and after (10, 20, 30, and 40 min) the Hebbian+ protocol. The waveforms represent an average of 20 MEPs. *B*: the box plot chart shows the MEP size before and after the Hebbian+ protocol (*n* = 11 rats). The top and bottom lines of the box indicate the 75th percentile (top quartile) and 25th percentile (bottom quartile), respectively. The lines in the middle of the boxes indicate the 50th percentile (median). The two bars extend from the maximum and minimum values. The dot within the box indicates the arithmetical mean. The dotted horizontal line indicates the baseline MEP size. *C*: data from individual rats. The abscissa indicates the time at which measurements were taken (Baseline, 10, 20, 30, and 40 min after Hebbian+). The ordinate indicates the size of MEPs in the soleus muscle expressed at a percentage of the baseline. Error bars indicate the standard deviation. *P < 0.05.

Figure 3A illustrates raw MEP traces elicited by cortical electrical stimulation from a single representative rat, before and after a single session of Hebbian+ stimulation. Note that the MEP size increased in the soleus muscle 10, 20, 30, and 40 min after the stimulation. Repeated-measures ANOVA showed an effect of TIME (*F*_4,40_ = 9.5, *P* < 0.001, $\eta_{\text{p}}^{2}$ = 0.49) on MEP size. We found that after Hebbian+ (*n* = 11 rats), MEP size increased after 10 min (134.4 ± 31.3%, *P* = 0.005), 20 min (128.2 ± 23.1%, *P* = 0.003), 30 min (129.1 ± 20.5%, *P* < 0.001), and 40 min (131.1 ± 20.8%, *P* < 0.001) compared with baseline (Fig. 3B). The percentage increase in MEP size at the 10-min intervals after the stimulation period ranged from 28.3% to 34.4%, without a significant decrease over time. Notably, all animals showed an increase in MEP size at most times tested (Fig. 3C).

Repeated-measures ANOVA showed no effect of TIME (*F*_1.25,12.52_ = 0.48, *P* = 0.55) on EMG background. EMG background was similar between values at baseline (135.5 ± 100.0 µV), and after 10 min (138.7 ± 222.9 µV, *P* = 1.0), 20 min (151.7 ± 201.3 µV, *P* = 1.0), 30 min (110.6 ± 89.4 µV, *P* = 1.0), and 40 min (106.0 ± 86.5 µV, *P* = 0.33) after Hebbian+.

### Hebbian−

**Figure 4.**
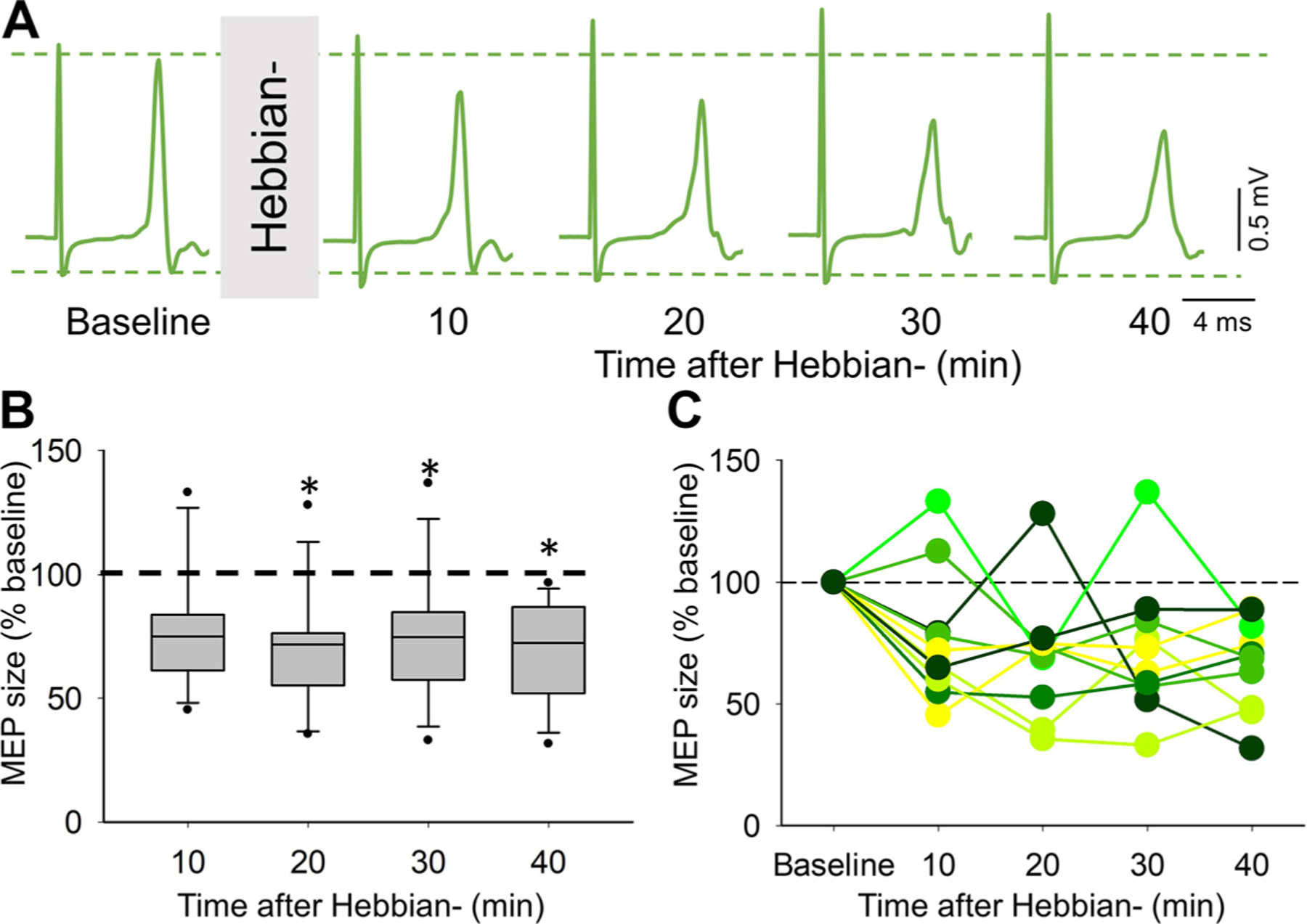
Hebbian− (*A*). Representative raw motor-evoked potential (MEP) traces from the soleus muscle in an awake adult rat recorded before (baseline) and after (10, 20, 30, and 40 min) the Hebbian− protocol. The waveforms represent an average of 20 MEPs. *B*: the box plot chart shows the MEP size before and after the Hebbian− protocol (*n* = 12 rats). The top and bottom lines of the box indicate the 75th percentile (top quartile) and 25th percentile (bottom quartile), respectively. The lines in the middle of the boxes indicate the 50th percentile (median). The two bars extend from the maximum and minimum values. The dot within the box indicates the arithmetical mean. The dotted horizontal line indicates the baseline MEP size. *C*: data from individual rats. The abscissa indicates the time at which measurements were taken (Baseline, 10, 20, 30, and 40 min after Hebbian−). The ordinate indicates the size of MEPs in the soleus muscle expressed at a percentage of the baseline. Error bars indicate the standard deviation. *P < 0.05.

Figure 4A illustrates raw MEP traces elicited by cortical electrical stimulation from a single representative rat (the same animal shown with Hebbian+ stimulation in Fig. 3), before and after a single session of Hebbian− stimulation. Note that in this rat, MEP size decreased in the soleus muscle 10, 20, 30, and 40 min after the stimulation. Repeated-measures ANOVA showed an effect of TIME (*F*_4,44_ = 5.40, *P* = 0.001, $\eta_{\text{p}}^{2}$ = 0.33) on MEP size. We found that, after Hebbian− stimulation (*n* = 12 rats), MEP size decreased after 10 min (77.8 ± 24.5, *P* = 0.02), 20 min (69.6 ± 23.4, *P* = 0.003), 30 min (73.8 ± 25.7, *P* = 0.01), and 40 min (69.7 ± 19.4, *P* < 0.001) compared with baseline (Fig. 4B). The percentage decrease in MEP size at the 10-min intervals after the stimulation period ranged from 22.2% to 30.3%, without a significant change over time. Notably, all animals showed a decrease in MEP size at most times tested (Fig. 4C). Repeated-measures ANOVA showed no effect of TIME (*F*_1.99,21.83_ = 0.43, *P* = 0.66) on EMG background. EMG background was similar across time (baseline = 132.9 ± 113.0 µV, after 10 min = 139.9 ± 133.1 µV, *P* = 1.0, 20 min = 136.9 ± 154.7 µV, *P* = 1.0, 30 min = 112.8 ± 118.4 µV, *P* = 1.0, 40 min = 116.6 ± 139.2 µV, *P* = 1.0).

### Hebbian+ and Hebbian− Effects in Rats from Different Surgeons and Sex

**Figure 5.**
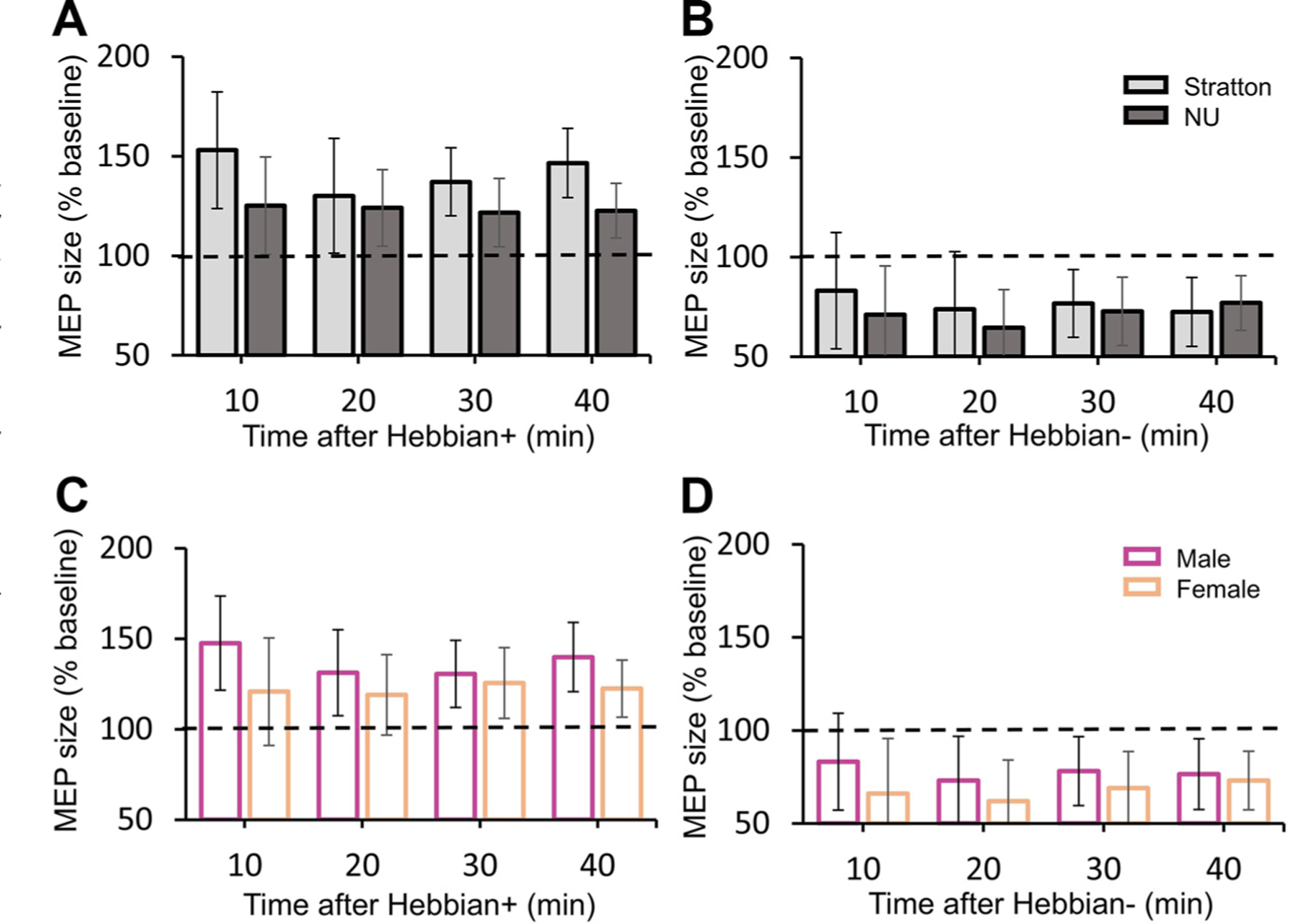
Hebbian+ and Hebbian− effects in rats performed by different surgeons and sex. The graphs show comparisons of average motor-evoked potential (MEP) size after Hebbian+ (5 Stratton rats, 6 NU rats) and Hebbian− (5 Stratton rats, 7 NU rats) protocols from rats performed by different surgeons (*A* and *B*: Stratton = light gray, NU = dark gray) or sex (*C* and *D*; males = magenta, females = orange) (7 male rats, 4 female rats for Hebbian+ and 7 male rats, 5 female rats for Hebbian−). In all graphs, the abscissa indicates the time at which measurements were taken (Baseline, 10, 20, 30, and 40 min after Hebbian−). In all graphs, the ordinate indicates the size of MEPs in the soleus muscle expressed at a percentage of the baseline. No significant differences were found between rats performed by different surgeons or different sex. Error bars indicate the standard deviation.

Repeated-measures ANOVA showed an effect of TIME (*F*_4,36_ = 9.76, *P* < 0.001, $\eta_{\text{p}}^{2}$ = 0.52), but not SURGEONS (*F*_1,9_ = 1.62, *P* = 0.24), nor in their interaction (*F*_4,36_ = 0.85, *P* = 0.50) on MEP size after Hebbian+ (Fig. 5A). Similarly, split plot repeated-measures ANOVA showed an effect of TIME (*F*_4,40_ = 5.54, *P* = 0.001, $\eta_{\text{p}}^{2}$ = 0.36), but not SURGEONS (*F*_1,10_ = 0.20, *P* = 0.67), nor in their interaction (*F*_4,40_ = 1.48, *P* = 0.23) on MEP size after Hebbian− (Fig. 5B). Note that we did not find significant differences in the MEP size at any time after ending the protocols between the two SURGEONS groups.

Repeated-measures ANOVA showed an effect of TIME (*F*_4,36_ = 7.51, *P* < 0.001, $\eta_{\text{p}}^{2}$ = 0.46), but not SEX (*F*_1,9_ = 1.05, *P* = 0.33) nor in their interaction (*F*_4,36_ = 0.77, *P* = 0.55) on MEP size after Hebbian+ (Fig. 5C). Repeated-measures ANOVA also showed an effect of TIME (*F*_4,40_ = 5.38, *P* = 0.001, $\eta_{\text{p}}^{2}$ = 0.35), but not SEX (*F*_1,10_ = 1.14, *P* = 0.31), nor in their interaction (*F*_4,40_ = 0.89, *P* = 0.48) on MEP size after Hebbian− stimulation (Fig. 5D).

### Correlation

**Figure 6.**
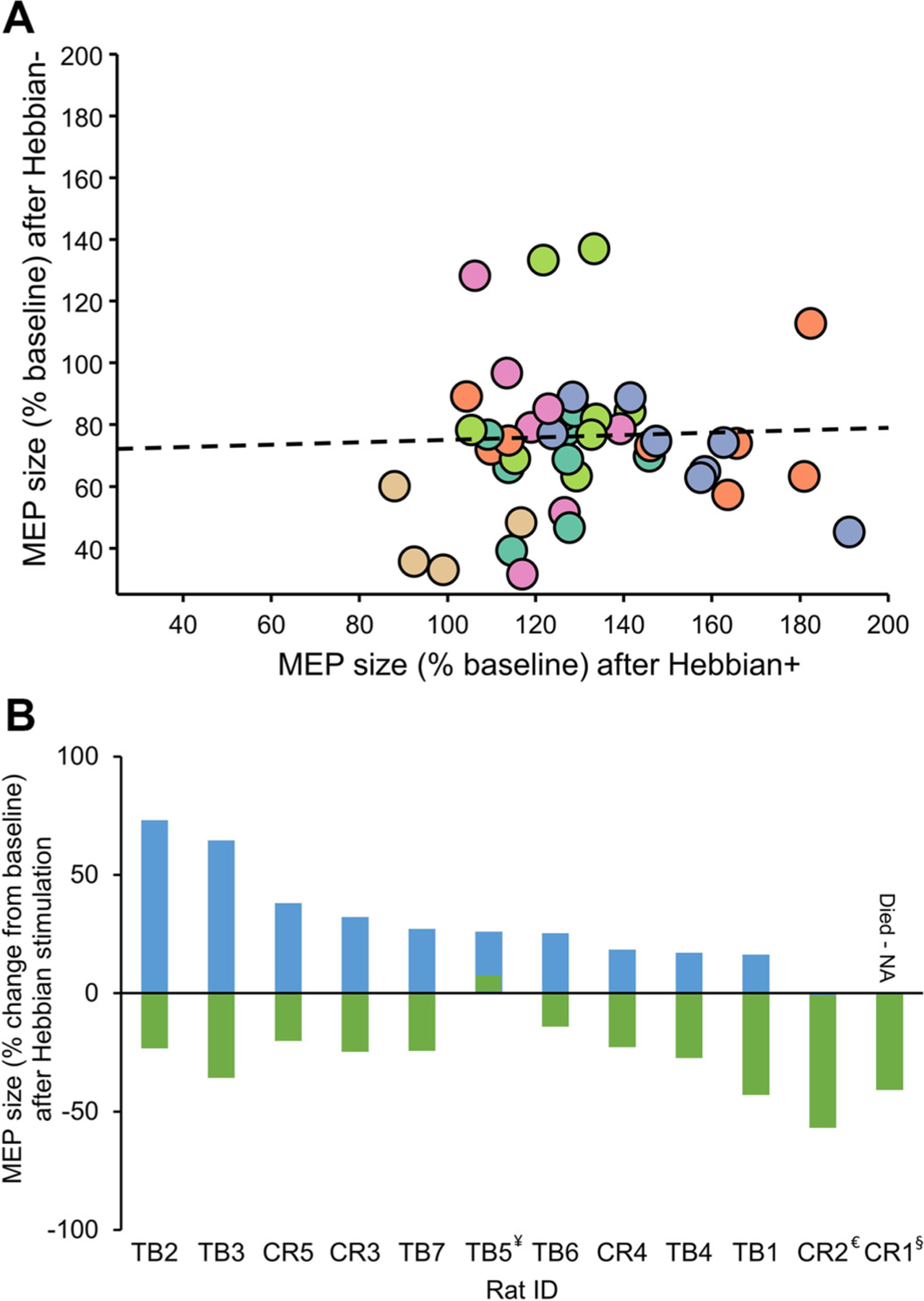
Correlation. The graphs display individual data from rats. *A*: each rat’s motor-evoked potential (MEP) size after Hebbian+ stimulation on the *x*-axis (expressed as a percentage of baseline MEP size) against the MEP size after Hebbian− stimulation on the *y*-axis (also as % of baseline) (*n* = 11 rats). *B*: the average MEP size at 10, 20, 30, and 40 min poststimulation for five rats from Taconic Biosciences (TB1-TB5, Taconic/Stratton rats) and seven rats from Charles River (CR1-CR7, Charles/NU rats). The plot shows each rat’s MEP size (expressed as the percent change from baseline) on the *y*-axis, whereas the *x*-axis identifies individual rats, ordered according to the degree of MEP facilitation in the Hebbian+ protocol. ¥, TB5 showed average facilitation of 5.25% following Hebbian−, §, CR1 died before Hebbian+, and €, CR2 showed an inhibition of −1.03% following Hebbian+.

Figure 6 illustrates the correlation of MEP size between the Hebbian+ and Hebbian− stimulation protocols. Pearson’s correlation analysis revealed no significant relationship between MEP sizes following Hebbian+ and Hebbian− stimulation (*r* = 0.055, *P* = 0.725). Although all animals demonstrated an increase in MEP size after Hebbian+ stimulation (Fig. 3C) and a decrease after Hebbian− stimulation (Fig. 4C) at most time points tested, averaging the MEP responses across all poststimulation time points revealed that only one rat (CR2) did not show MEP increases following the Hebbian+ protocol, and another rat (TB5) did not exhibit MEP suppression after the Hebbian−− protocol (Fig. 6B), highlighting the overall robustness of these findings.

## DISCUSSION

For the first time, we present evidence from awake rats showing that paired stimulation, applied according to Hebbian STDP principles, can bidirectionally modulate the size of MEPs in the soleus muscle. Individualized timing for each animal was determined based on MEP, F-wave, H-reflex, and M-max latencies, using chronically implanted epidural cortical and peripheral nerve electrodes. These latencies enabled precise timing of central volleys to arrive either 2.5 ms before (Hebbian+) or 15 ms after (Hebbian−) peripheral volleys, as dictated by the respective protocols. Our findings show that the Hebbian+ protocol led to an average 30% increase in MEP size, persisting for at least 40 min poststimulation, whereas the Hebbian− protocol resulted in a 27% decrease, with effects lasting similarly. Notably, results were consistent across study sites and between male and female rats. Given the established efficacy of Hebbian+ protocols in enhancing sensorimotor function in individuals with SCI (8, 10), our rat model of Hebbian stimulation provides a valuable platform for investigating experimental combination therapies aimed at further improving motor recovery following SCI and other neurological disorders.

Previous studies have attempted to investigate the effects of paired stimulation or paired associative stimulation in the spinal cord of rats; however, the results remain challenging to interpret (11–17). These investigations have produced mixed findings, with some reporting that repeated paired pulses increased MEP size or lowered excitation thresholds (11, 13–17), whereas others observed no significant changes (12). Importantly, none of these studies have demonstrated a reversible facilitatory and inhibitory effect on MEP size as suggested by Hebbian timing rules and STDP principles (11–17). Factors contributing to this variability may include the lack of precise estimations of CCT and PCT (11–17), the use of trains of pulses at different stimulation sites (11, 13–17), and the administration of anesthetics during experiments (11, 14, 16). To address these issues, we developed a model in which paired stimulation was applied to awake, unrestrained adult rats using Hebbian timing rules, guided by electrophysiological measurements of CCT and PCT in individual animals, and without the use of anesthesia. This was achieved using chronically implanted epidural screw electrodes over the cortex and a cuff electrode around the peripheral nerve. Our awake rat model for Hebbian stimulation offers the added advantage that the stimulation protocol can be repeated over multiple consecutive days without the need for daily anesthesia.

There are two key points that are critical to discuss. The first critical question concerns which descending motor pathways were targeted during our Hebbian protocols. Current evidence indicates that direct monosynaptic connections between corticospinal neurons and spinal motoneurons are sparse or absent in adult rats (29, 30). In contrast, the reticulospinal tract forms direct monosynaptic connections with lumbar motoneurons, as demonstrated by excitatory postsynaptic potential (EPSP) studies in rats (31, 32). Our results suggest that it is likely that reticulospinal neurons contributed to the observed effects. This is supported by the short MEP latencies (6–7 ms) recorded in the soleus muscle, which are too brief to be explained by conduction through the corticospinal tract, given its relatively low conduction velocity (29, 33) compared with reticulospinal tract (34). This finding aligns with lesion studies showing that dorsal column lesions—which disrupt most corticospinal fibers—markedly reduced MEPs at longer latencies (8–12 ms and 14–18 ms), whereas ventral column lesions abolished the shortest latency MEPs (4–6 ms), implicating ventrally descending motor pathways (35). The reticulospinal tract is the most likely candidate for these ventral pathways. Further supporting this, prior research indicates that electrical stimulation of the corticospinal tract in the brainstem evokes early EPSPs primarily via activation of the reticulospinal tract through corticospinal collaterals (29). Thus, it is plausible that some of the short-latency MEPs observed in our study are mediated by a similar indirect pathway. Although invasive electrical stimulation with large electrodes and high currents, as used here, limits precise selectivity for specific descending tracts, the MEP latency profiles and lesion data collectively suggest that reticulospinal tract activation plays a role in the responses we observed.

A second critical question concerns the ISIs used during Hebbian stimulation and how they compare to human studies. Research in humans with chronic SCI has demonstrated that Hebbian+ and Hebbian− protocols enhance and suppress MEP size, respectively, in leg muscles after ~200 paired pulses (27). Consistent with these findings, we observed an increase in MEP size after Hebbian+ and a decrease after Hebbian− in all rats tested. Notably, in our rat model, we applied ISIs that have been successfully used in humans (6, 7). In the Hebbian+ protocol, the ISI ensured that descending volleys arrived at the presynaptic terminal of descending neurons 2.5 ms before antidromic volleys reached motoneuron dendrites. Conversely, in the Hebbian− protocol, the ISI allowed antidromic volleys to reach motoneuron dendrites 15 ms before descending volleys reached the presynaptic terminal. A key question is how these same ISIs can be effective in both humans and rats, given the differences in size and neural architecture across species. In animal models, a narrow transition zone of around 5 ms has been reported between potentiation and depression (4). Indeed, protocols in rats using this narrow interval have shown increased MEP size after repeated paired stimulation (11, 13). In contrast, studies in rats (12) and humans (6) employing a broader interval to elicit facilitation were unsuccessful in increasing MEP size. Similarly, in humans (6, 7) and in our rat model, volleys arriving at motoneurons ~15 ms before presynaptic arrival resulted in MEP suppression, suggesting that the excitatory and inhibitory effects of Hebbian STDP protocols may follow a conserved temporal plasticity window across species (36). Multiple circuits can converge onto a common target, but the rules governing this convergence remain poorly understood. Thus, the results from existing paired associative stimulation protocols in rats align more closely with a convergent model of plasticity (18). In our study, the reversible effects of our Hebbian stimulation protocols on MEP size observed after 180 paired stimuli at 0.1 Hz that lasted up to 40 min, and in some animals for up to 120 min, suggest that we engaged, at least to some extent, STDP principles. These findings are consistent with timing-dependent plasticity changes reported in other animal studies (4, 5). Although we did not record directly at the synapse, we were able to generate precise estimates of action potential arrival times through electrophysiological measurements, stimulating different levels of the descending pathway in each animal.

### Methodological Considerations

Our results demonstrate that it is feasible to reliably determine the latencies of MEPs, M-max, and H-reflex, which are vital for controlling the ISI in the Hebbian+ and Hebbian− protocols (8, 9, 27, 37). As in human studies, we estimated CCT and PCT by calculating latencies from electrophysiological measurements. This approach accounts for the possible involvement of both direct and indirect descending motor pathways to spinal motoneurons (38). MEPs, M-max, and H-reflex were recorded in rats from *week 3* up to 22 wk postsurgery. The average MEP latency elicited in the soleus muscle by cortical stimulation was found to be 6.3 ± 0.4 ms, which agrees with reported MEP latency ranges in the gastrocnemius muscle in adult Sprague–Dawley rats (39, 40). Similarly, the measured M-wave latency of 1.3 ± 0.2 ms and H-reflex latency of 6.1 ± 1.0 ms both fall within the reported ranges for adult Sprague–Dawley rats (41, 42). These findings confirm the integrity of the peripheral motor and reflex pathways studied in our animal model. It is possible that during the first weeks after surgery, the natural inflammatory response at the wound sites affected the generation and/or recording of electrophysiological responses. The stainless-steel electrodes, which are commonly used in rodents (11, 13–15, 17), may have contributed to local inflammation (43). It is likely that inflammation had a lesser impact on our outcomes, as measurements were taken 3 to 10weeks postsurgery—a period during which electrophysiological responses were observed in all animals.

Although current Hebbian+ protocols in humans with SCI primarily aim to target plasticity at corticospinal-moto-neuronal synapses (7–10), it is likely that multiple descending pathways contribute to improvements in various aspects of hand and leg function. Indeed, human studies have demonstrated that STDP-like plasticity effects can also be induced in the reticulospinal tract (44). This suggests that strengthening multiple descending motor pathways, such as corticospinal and reticulospinal pathways through Hebbian plasticity, may drive broader beneficial plasticity. The observation that combining Hebbian+ protocols with exercise (skill-specific practice) further enhances recovery supports this idea, as practice appears to encourage and shape the plasticity enabled by improved corticospinal control (45). A similar interaction between targeted plasticity and practice seen after Hebbian protocols (8–10) is also observed with H-reflex operant conditioning—therapy that modulates plasticity in a specific central nervous system (CNS) pathway and has been shown to facilitate recovery in individuals with SCI (46, 47).

While Hebbian-mediated recovery of function in individuals with SCI is promising, its effects remain limited, particularly in those with more severe paralysis—specifically, individuals with anatomically incomplete injuries but no voluntary movement. To address this need, we aimed to translate the clinically successful Hebbian+ protocol into an adult rat model. The model described and validated in this study serves as a valuable tool for elucidating the mechanisms underlying this protocol’s success and for refining its parameters to maximize its therapeutic potential.

## Figures and Tables

**Table 1.** Overview of the 12 rats involved in the study

Rat ID	Weight at Surgery, g	Sex (M/F)	Start MEP (wpi)	Hebbian +/− Protocol (wpi)	MEP Recording Length (wpi)	Rat Strain/Vendor
TB1	430	M	3	3/3	NR	SD/TB
TB2	426	M	3	3/3	NR	SD/TB
TB3	368	M	7	7/7	NR	SD/TB
TB4	355	M	4	4/4	NR	SD/TB
TB5	400	M	4	4/4	NR	SD/TB
CR1	296	F	4	NR/10	10	SD/CR
CR2	285	F	5	9/9	15	SD/CR
CR3	255	F	4	9/8	16	SD/CR
CR4	255	F	5	7/7	20	SD/CR
CR5	285	F	5	10/10	22	SD/CR
TB6	359	M	3	8/8	13	SD/TB
TB7	319	M	4	4/3	NR	SD/TB

Five rats performed at Stratton (TB1–TB5, from Taconic Biosciences, Stratton rats) and seven rats performed at Northwestern University (CR1–CR5, from Charles River Laboratories, TB6–TB7 from Taconic Biosciences, NU rats) were implanted with central and peripheral electrodes to implement Hebbian+ and Hebbian− stimulation protocols. TB, Taconic Biosciences; CR, Charles River Laboratories; F, female; g, grams; M, male; wpi, weeks postimplantation; Hebbian+, Facilitatory Hebbian stimulation; Hebbian−, Inhibitory Hebbian stimulation; NR, not recorded; SD, Sprague–Dawley; Start MEP, week at which motor-evoked potential was tested for the first time after implantation; MEP recoding length, week at which motor-evoked potential was tested for the last time after implantation. This metric was tested in NU rats as an indicator of electrode stability over time.

## Data Availability

Data will be made available upon reasonable request.
